# Loss of NAC1 Expression Is Associated with Defective Bony Patterning in the Murine Vertebral Axis

**DOI:** 10.1371/journal.pone.0069099

**Published:** 2013-07-26

**Authors:** Kai Lee Yap, Polina Sysa-Shah, Brad Bolon, Ren-Chin Wu, Min Gao, Alice L. Herlinger, Fengying Wang, Francesco Faiola, David Huso, Kathleen Gabrielson, Tian-Li Wang, Jianlong Wang, Ie-Ming Shih

**Affiliations:** 1 Department of Pathology, Johns Hopkins Medical Institutions, Baltimore, Maryland, United States of America; 2 Department of Molecular and Comparative Pathobiology, Johns Hopkins University, Baltimore, Maryland, United States of America; 3 Department of Veterinary Biosciences, the Ohio State University, Columbus, Ohio, United States of America; 4 Biotechnology Program/RENORBIO, Health Sciences Center, Federal University of Espirito Santo, Vitória, Brazil; 5 Department of Developmental and Regenerative Biology, Black Family Stem Cell Institute, Mount Sinai School of Medicine, New York, New York, United States of America; 6 Departments of Gynecology/Obstetrics, Johns Hopkins Medical Institutions, Baltimore, Maryland, United States of America; National Cancer Institute, United States of America

## Abstract

NAC1 encoded by *NACC1* is a member of the BTB/POZ family of proteins and participates in several pathobiological processes. However, its function during tissue development has not been elucidated. In this study, we compared homozygous null mutant Nacc1^-/-^ and wild type Nacc1^+/+^ mice to determine the consequences of diminished NAC1 expression. The most remarkable change in Nacc1^-/-^ mice was a vertebral patterning defect in which most knockout animals exhibited a morphological transformation of the sixth lumbar vertebra (L6) into a sacral identity; thus, the total number of pre-sacral vertebrae was decreased by one (to 25) in Nacc1^-/-^ mice. Heterozygous Nacc1^+/-^ mice had an increased tendency to adopt an intermediate phenotype in which L6 underwent partial sacralization. Nacc1^-/-^ mice also exhibited non-closure of the dorsal aspects of thoracic vertebrae T10-T12. Chondrocytes from Nacc1^+/+^ mice expressed abundant NAC1 while Nacc1^-/-^ chondrocytes had undetectable levels. Loss of NAC1 in Nacc1^-/-^ mice was associated with significantly reduced chondrocyte migratory potential as well as decreased expression of matrilin-3 and matrilin-4, two cartilage-associated extracellular matrix proteins with roles in the development and homeostasis of cartilage and bone. These data suggest that NAC1 participates in the motility and differentiation of developing chondrocytes and cartilaginous tissues, and its expression is necessary to maintain normal axial patterning of murine skeleton.

## Introduction

Nucleus accumbens-associated protein 1 (NAC1) encoded by *NACC1* belongs to the Bric-a-Brac Tramtrack Broad complex /Pox virus and Zinc finger (BTB/POZ) family. This molecule mediates protein homo- or hetero-dimerization through its BTB domain to form higher-order transcription complexes [1]. NAC1 has emerged as a molecule that plays an important role in several pathobiological processes. *NACC1* was discovered as one of the upregulated genes in the rat nucleus accumbens after acute cocaine treatment [2,3]. Induction of NAC1 expression in the murine nucleus accumbens was subsequently demonstrated to modulate long-term behavioral and neurochemical responses to psychomotor stimulants [4] and to be essential for the translocation of the ubiquitin-proteasome system (UPS) from the nucleus into dendritic spines of cortical neurons [5]. NAC1 also was found to maintain the proliferative capacity and stemness of mouse embryonic stem cells [6] by acting in association with homeobox protein Nanog and other nuclear factors [7]. In human cancers, *NACC1* upregulation is associated with disease aggressiveness, development of resistance to chemotherapeutic agents, and tumor recurrence in ovarian, endometrial, and cervical carcinomas [8–16]. Moreover, analysis of The Cancer Genome Atlas (TCGA) ovarian cancer data revealed that *NACC1* is one of the top genes that shows a significant positive correlation between DNA and RNA copy number [15,17], indicating that NAC1 is a potential “driver” in promoting cancer development through multiple mechanisms related to transcription-dependent and -independent pathways. Specifically, abundant NAC1 protein is essential for cancer cells to complete cytokinesis [18], promote cancer cell migration and motility [14,19], maintain cellular survival [9,11], prevent cellular senescence [20], and activate autophagy by collaborating with the high-mobility group protein B1 (HMGB1) pathway in the presence of cisplatin [21].

In view of the important roles of NAC1 in mouse stem cell biology, in nucleus accumbens-related addictive behaviors, and in human cancer pathogenesis, it seems likely that NAC1 also will have essential roles in guiding the development of normal tissues. It has been reported that the mouse *Nacc1* gene, the homolog of human *NACC1*, is expressed in various normal murine tissues, especially in the central nervous system (CNS) where NAC1 functions as a co-repressor for BTB proteins in mature animals [22,23]. Therefore, in this study, we examined the phenotypes of *Nacc1* homozygous knockout (Nacc1^-/-^) and heterozygous (Nacc1^+/-^) knockout mice compared to their wild type (Nacc1^+/+^) littermate controls to investigate the hypothesis that decreased NAC1 expression will affect regulation of embryonic development and tissue homeostasis. We found that Nacc1^-/-^ mice are not embryonic lethal and lack grossly noticeable morphological phenotypes, but they do exhibit a slight survival disadvantage and suffer from a significant defect in patterning of the axial skeleton.

## Materials and Methods

### Mouse husbandry and production

Ethics statement: Mice were housed and handled according to the specified approved protocol (MO09M473) and guidelines of the Johns Hopkins University Animal Care and Use Committee.

Breeding pairs were housed in filter-capped microisolator cages and fed a commercial rodent chow and filter-purified tap water *ad libitum*. Animals were maintained on a cycle of 12-hr alternating light and dark periods at 22 ± 1°C and relative humidity of 40 ± 10%.

**Figure 1:**
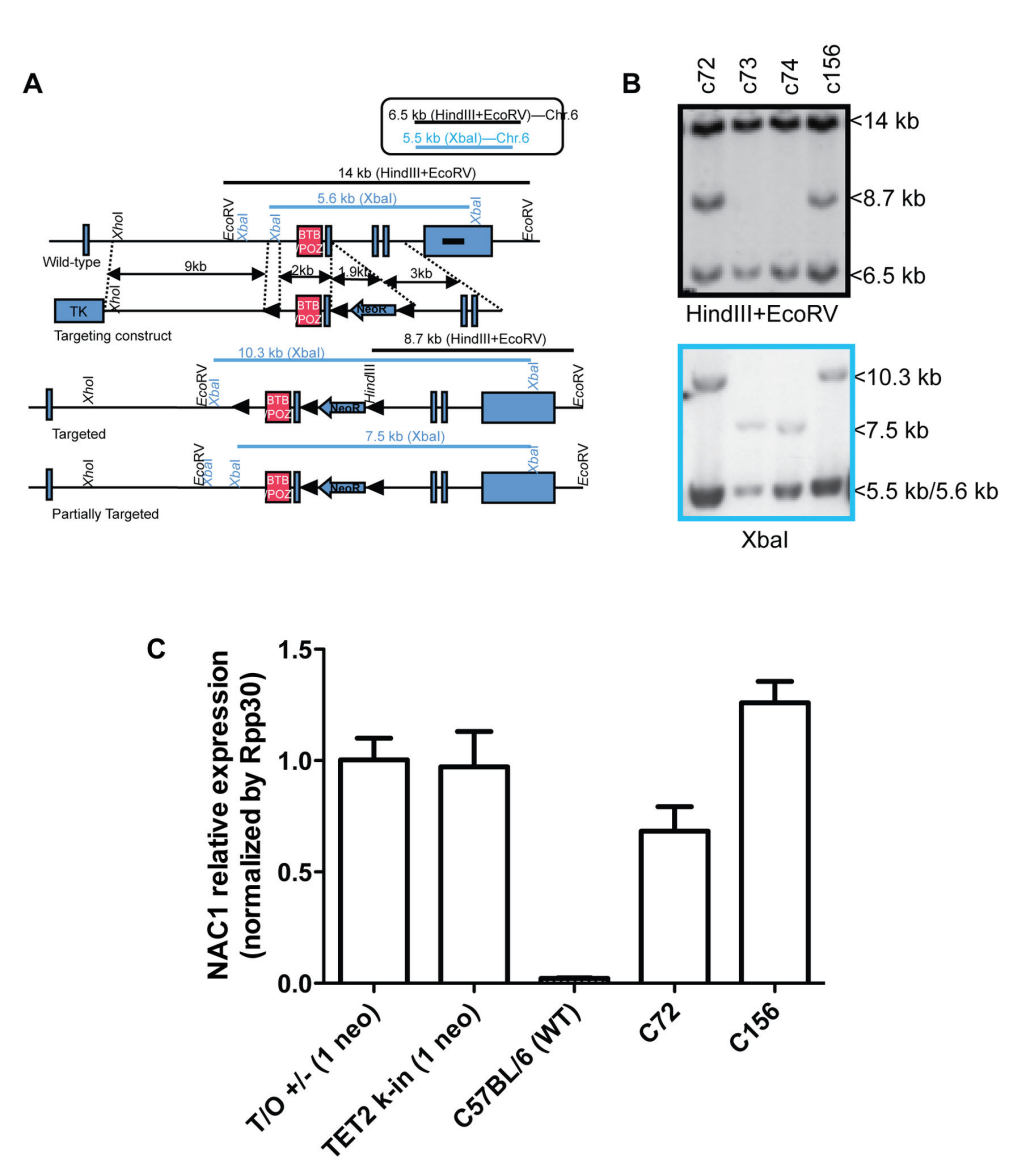
Strategy used in generation of *Nacc1* knockout allele. **A**. Schematic illustration of gene targeting and Southern strategies. Black lines and blue lines indicate the expected sizes of fragments detected by Southern hybridization of genomic DNAs digested by HindIII/EcoRV and XbaI, respectively. The black bar indicates the probe for Southern hybridization. The fragments within the square on the top are the expected bands from cross-hybridization of the probe to the genomic DNA in Chr. 6. Note *Nacc1* is located in Chr. 8. **B**. Representative clones from Southern hybridization as shown in panel A. Two positive clones, c72 and c156, were identified. Clones c73 and c74 are partially targeted because they lack the first LoxP site as shown in panel A. **C**. Quantitative real-time PCR to measure neo levels revealed that both positive clones, c72 and c156, likely have a single copy insertion of the neo cassette. The housekeeping gene control used in this experiment was *Rpp30*, and the data was normalized to single neo copy controls (T/O +/- and TET2 k-in) to calculate relative neo levels.

To produce a floxed allele of the *Nacc1* gene, a targeting vector was constructed in which exons 2 and 3 encompassing the BTB-POZ domain of the NAC1 protein were flanked by two loxP sites and a phosphoglycerokinase (PGK)-neomycin resistance (*neo*) cassette (Figure 1A). The linearized targeting vector was electroporated into CJ7 embryonic stem (ES) cells. Eight of the 227 G418- and ganciclovir-resistant ES cell colonies screened were shown to be homologous recombinants by Southern blot analysis (two of them, c72 and c156, were shown in Figure 1B). Two clones (c73 and c74) were found to be partially targeted resulting in the lack of the first LoxP site (Figure 1A bottom and Figure 1B). One of the positive clones was injected into blastocysts of 129 genetic background to generate chimeras, which were bred with C57B/L6 to test germ line transmission and generate F1 *Nacc1* ^+/flox^ mice. To test that the positive clones were due to a single neo cassette insertion event, we conducted a realtime quantitative PCR assay with neo and *Rpp30* primers as housekeeping control [5], to measure neo levels in known controls and the clones and validated that the selected clones were single insertion events (Figure 1C).

The primers used for this assay are as follows:

mRpp30-F: 5’-TCCAGTGTGCAAGAAAGCTAAATG-3’
mRpp30-R: 5’- GGCAGTGCGTGGAGACTCA-3’
mNeo-F: 5’- CAATAGCAGCCAGTCCCTTC-3’
mNeo-R: 5’- AGACAATCGGCTGCTCTGAT-3’

These *Nacc1* ^+/flox^ mice were further bred with Gata1Cre to generate Nacc1^+/-^ progenies for further studies. Genotyping of mice was conducted at 3 weeks of age by collecting tail snips and extracting DNA by boiling in 50 mM NaOH for 10 minutes followed by neutralization with Tris-HCl buffer before using an aliquot for polymerase chain reaction (PCR) analysis. Primer sequences for PCR genotyping were as follow.

#1NacKO-F: 5'-CTGGGGAATGGATGGTTTTA-3';
#3RRO154-F: 5’-TGAGAAGGTAGAGGCCCTTCC-3’;
#5NacKOnull-R: 5'-CAGGGGCTGACAGTCATCTT-3'.

Using the specified primers, the PCR product size from the *Nacc1* knockout allele is 40 base pairs smaller than the PCR product of the *Nacc1* wild-type allele.

Mendelian ratios of breeding outcomes were calculated according to the weaning age of 3 weeks. A G-test for goodness of fit was conducted to assess whether or not the Mendelian ratio had deviated by a statistically significant degree from the expected 1:2:1 ratio from Nacc1^+/-^ mate pair breedings.

### Alizarin red staining of bone in the adult murine skeleton

Alizarin red staining of the adult murine skeleton was accomplished according to a published protocol [24], with slight modifications. Briefly, mice were euthanized, eviscerated and soaked in acetone overnight to remove fat. Specimens were transferred to 2% potassium hydroxide (KOH) to clear the soft tissues. After 2-3 days, specimens were transferred to the staining solution containing 0.005% Alizarin red (Sigma Aldrich) and 1% KOH. After overnight incubation at room temperature, specimens were transferred to 2% KOH for de-staining and additional pre-clearing of soft tissues. The stained specimens were photographed using a Nikon stereomicroscope with trans-illuminated light source.

### Alcian blue staining of cartilage in the embryonic mouse skeleton

Alcian blue staining of cartilage in late-gestation mouse embryos was performed as described [24]. Briefly, mouse embryos at later embryonic stages were dissected from the pregnant dams and fixed in Bouin’s solution for 2 hours. Next, embryos were incubated with 0.1% ammonium hydroxide in 70% ethanol; the solution was changed at intervals until it remained clear. The embryos were then equilibrated in 5% acetic acid for 1 hour before being transferred to the staining solution containing 0.05% Alcian blue 8GX (Sigma Aldrich), 5% acetic acid and 70% ethanol for 2 hours. The embryos next were de-stained in 5% acetic acid / 70% ethanol and transferred to the BABB solution (Benzyl alcohol: Benzyl Benzoate, 1:2) to clear soft tissues. The stained embryos were photographed as described above.

### Immunohistochemistry for NAC1 expression

Embryonic day 16 (E16) mouse embryos were harvested from pregnant dams, fixed in Bouin’s solution for 2 hours at room temperature, then transferred to 70% ethanol. The ethanol was changed regularly until it remained clear. Embryos were dehydrated in graded ethanol solutions (85%, 95%, 100%) and xylene, embedded in paraffin, and sectioned at 5µm for immunohistochemistry (IHC) processing. Formalin-fixed and paraffin-embedded human embryonic tissues (6-10 weeks of gestation) were retrieved from abortus cases in the Department of Pathology at the Johns Hopkins Hospital. Sections were deparaffinized in xylene and graded ethanol solutions and placed in citrate-based buffer (pH 7.4; Trilogy pretreatment solution, Cell Marque Corporation, USA). Antigen retrieval for slides of both mouse and human origin was accomplished by heating the slides in Trilogy buffer for 15 minutes using a low-power (1000 W) microwave (Panasonic) (setting power level 5) followed by peroxidase blocking using 3% H_2_O_2_ for 5 min at room temperature. The slides then were blocked with 10% fetal bovine serum (FBS) in 1% Tween-TBS (TBST) for at least 30 minutes at room temperature before incubation at 4°C overnight with a primary anti-NAC1 antibody (diluted 1:200 in 1% BSA-PBS for rabbit polyclonal anti-mouse NAC1 IgG, Abcam; diluted 1:500 in 1% BSA-PBS for mouse monoclonal anti-human NAC1 IgG human, Novus Biological). A suitable species-specific, horseradish peroxidase-conjugated secondary antibody (Dako) was applied for 1hr at RT to detect primary antibodies. Diaminobenzidine (DAB, Dako) was applied as the chromogen to visualize the location of the immunobridges. The incubation time of antibody and DAB was the same for both control and knockout mice to enable a fair comparison of expression levels. The slides were counterstained with hematoxylin.

### Primary culture of chondrocytes

Primary cultures of mouse chondrocytes were established as described [25]. Briefly, pups were euthanized on postnatal day 1 (P1), and costal cartilage was dissected and rinsed in phosphate-buffered saline (PBS, pH 7.4). The costal cartilage is of hyaline type that serves to prolong the ribs forward. connecting to the sternum and was chosen because it can provide a high number of chondrocytes for the migration and proliferation studies. The cartilage pieces were incubated for 90 minutes at 37°C in Dulbecco’s modified essential medium (DMEM) containing 10% FBS and collagenase A (3 mg/ml); (Roche, 11088793001) in DMEM to dissociate large amounts of soft tissue. Cartilage pieces then were incubated with collagenase A (0.5 mg/ml) in DMEM (10% FBS) at 37°C overnight to remove remaining soft tissue fragments. The cartilage pieces were transferred to fresh collagenase-free DMEM and dissociated by vigorous pipetting for 1 minute to generate single cell suspensions. The cells were added to the dishes at a density of 1 x 10^6^ cells/ml, grown for 2 days and used for experiments before they lost chondrocyte phenotypes. Chondrocytes rapidly differentiate and lose their characteristic morphologies and function after a prolonged culture. To verify the chondrocyte phenotype, the chondrocyte-derived proteoglycans in the extracellular matrix were stained with Alcian blue according to a published protocol [25].

### Real-time quantitative PCR to assess mRNA expression in chondrocytes

RNA was extracted from cultured mouse chondrocytes using the RNAeasy Plus kit (Qiagen), and reverse transcribed into cDNA (iScript cDNA kit, Biorad). Real-time quantitative PCR (qPCR) was carried out to detect the expression of the following genes including *Nacc1*, *Nacc2*, *Acan* (aggrecan), *Col1a2* (collagen 1a2), and *Col2a1* (collagen 2a1). Three replicate readings were collected for each data point, and the experiment was replicated twice. The results of the qPCR analysis were normalized to the expression of a constitutively expressed housekeeping gene, glyceraldehyde 3-phosphate dehydrogenase (*Gapdh*). The following were the forward (F) and reverse (R) primers for the murine (m) gene sequences:

*mNacc1*-F, 5'-TCAGAGTCCTGTAGCGCAGA-3';
*mNacc1*-R, 5'-GGCTGAGGCATCCGGTTAAG-3';
*mNacc2*-F, 5’- TGCTGCCTATGTCACGTCTC -3’;
*mNacc2*-R, 5’-GGCTCTGGTTTCTCTTGCAC-3’;
*mAcan*-F, 5'-CCTGCTACTTCATCGACCCC-3';
*mAcan*-R, 5'-AGATGCTGTTGACTCGAACCT-3';
*mCol1a2*-F, 5'-GGTGAGCCTGGTCAAACGG-3';
*mCol1a2*-R, 5'-ACTGTGTCCTTTCACGCCTTT-3';
*mCol2a1*-F, 5'-CAGGATGCCCGAAAATTAGGG-3';
*mCol2a1*-R, 5'-ACCACGATCACCTCTGGGT-3'.
*mGapdh*-F, 5'-AGGTCGGTGTGAACGGATTTG-3';
*mGapdh*-R, 5'- GGGGTCGTTGATGGCAACA-3'.

### Chondrocyte migration and proliferation assays

To evaluate cell migration, Transwell chambers (8 µM pores, polyethylene terephthalate membrane; BD Bioscience) were coated with fibronectin (5 µg/ml in PBS) for 2 hours at 37°C. The chambers were rinsed with PBS and air-dried until use. Mouse chondrocytes (1x10^5^ cells) of each *Nacc1* genotype were seeded into each top chamber in DMEM (10% FBS) and incubated at 37°C for 15 hours to allow migration to proceed; cellular migration for each genotype group was evaluated using four independent chamber replicates. At the end of the incubation period, cells on the top of the chamber were removed with a cotton swab, and the chambers were placed in 4% paraformaldehyde solution to fix the cells that had migrated through the membrane (i.e., onto the fibronectin-coated surface). The slides were immersed in a solution of 4', 6-diamidino-2-phenylindole (DAPI, 2 mg/ml; in PBS, Sigma), after which photomicrographs were taken. Cells were quantified in eight randomly chosen fields for each genotype group, where the fields were selected from among all four replicates.

For proliferation assays, approximately 3000 mouse chondrocytes of each genotype group were plated into each well of a 96-well plate and cultured in DMEM with 10% FBS. The medium was replaced by sodium dodecyl sulfate (SDS, 50 µl of 0.2% solution in PBS) at different time points. SYBR green I solution (150 µl of a 1:1000 dilution in water; Molecular Probes, Cat # S7567) was then added to each well and pipetted vigorously. Fluorescence intensity, which reflected the total cell number in each well, was assessed on a FLUOstar microplate reader (BMG Labtech). A proliferation curve was established for each genotype by calculating the fold-change in DNA content from Day 1 (i.e., the day on which the wells had been seeded with cells).

### Microarray gene expression profiling

RNAeasy Plus kit (Qiagen) was used to extract mRNA from primary cultures of mouse chondrocytes. Illumina MouseWG-6_v2 Expression BeadChip kit (Illumina) was used to profile the chondrocyte transcriptome derived from three Nacc1^-/-^ mice and two Nacc1^+/+^ (wild type) littermates. BeadArray data were pre-processed with log_2_ transformation followed by quantile normalization using the Bioconductor package as described [26]. Differential expression was assessed by an empirical Bayesian approach implemented in Bioconductor package [27]. Differential expression of the microarray gene candidates between the two genotypes was validated by qPCR. In addition, mRNA (same preparation that was used in microarray hybridization) was reversed transcribed into cDNA (iScript; BioRad) to analyze expression of four matrilin (*Matn*) genes. Three replicate readings were collected for each data point, and the experiment was repeated twice. The following primers were used:

*Matn1*-F, CAGGTCCCTGATAGCCTCAGT;
*Matn1*-R, CCACGGGTCTCACACTTCG;
*Matn2*-F, CTATGTATGCCGTTGGGGTAGG;
*Matn2*-R, AGCTTTTCACTTATTTCGCCCAT;
*Matn3*-F, TCTCCCGCATCATCGACACT;
*Matn3*-R, GTCGGAATAGGTGTTGAGCTG;
*Matn4*-F, GCCCCTTCGAGTTTGAGACC;
*Matn4*-R, ACGCTTTGCACTTGACTAGAAT.

### Statistical analysis

Statistical analyses were conducted using the Graphpad Prism software, and plotted accordingly. Statistical significance was determined using the indicated tests and expressed using the standard Michelin Guide scale (***p <0.001, **p <0.01 and *p <0.05).

## Results

### Loss of NAC1 decreases the number of viable mouse progeny

As the fertility of adult male and female Nacc1^-/-^ mice is low, Nacc1^+/-^ mating pairs were arranged to permit continuous reproduction of the Nacc1^-/-^ mice. Although viable Nacc1^-/-^ offspring were generated in Nacc1^+/-^ x Nacc1^+/-^ crosses, significantly fewer Nacc1^-/-^ mice were weaned (16.7%; G-test, * p < 0.05) than the number predicted by Mendelian genetics (25%), suggesting that *Nacc1* plays a role in prenatal and/or early postnatal development (Table 1). The significant shortfall of live Nacc1^-/-^ mice at weaning suggests incomplete penetrance of lethality in the NAC1-deficient offspring. As genotyping was undertaken at P21 (weaning), the exact timing of the lethal event could not be defined.

**Table 1:** Mendelian ratios of the outcome of Nacc1^+/-^ mice breeding.

**Genotype**	**Number of Mice (%)**
***Nacc1*^*+/+*^**	63 (28.5%)
***Nacc1*^*+/-*^**	121 (54.8%)
***Nacc1*^*-/-*^**	37 (16.7%)
**Total**	221 (100%)

G-test, p 0.01205357

* mice were accounted for at 3-weeks of age after weaning

### Loss of NAC1 expression is associated with a defect in axial skeleton patterning

**Figure 2:**
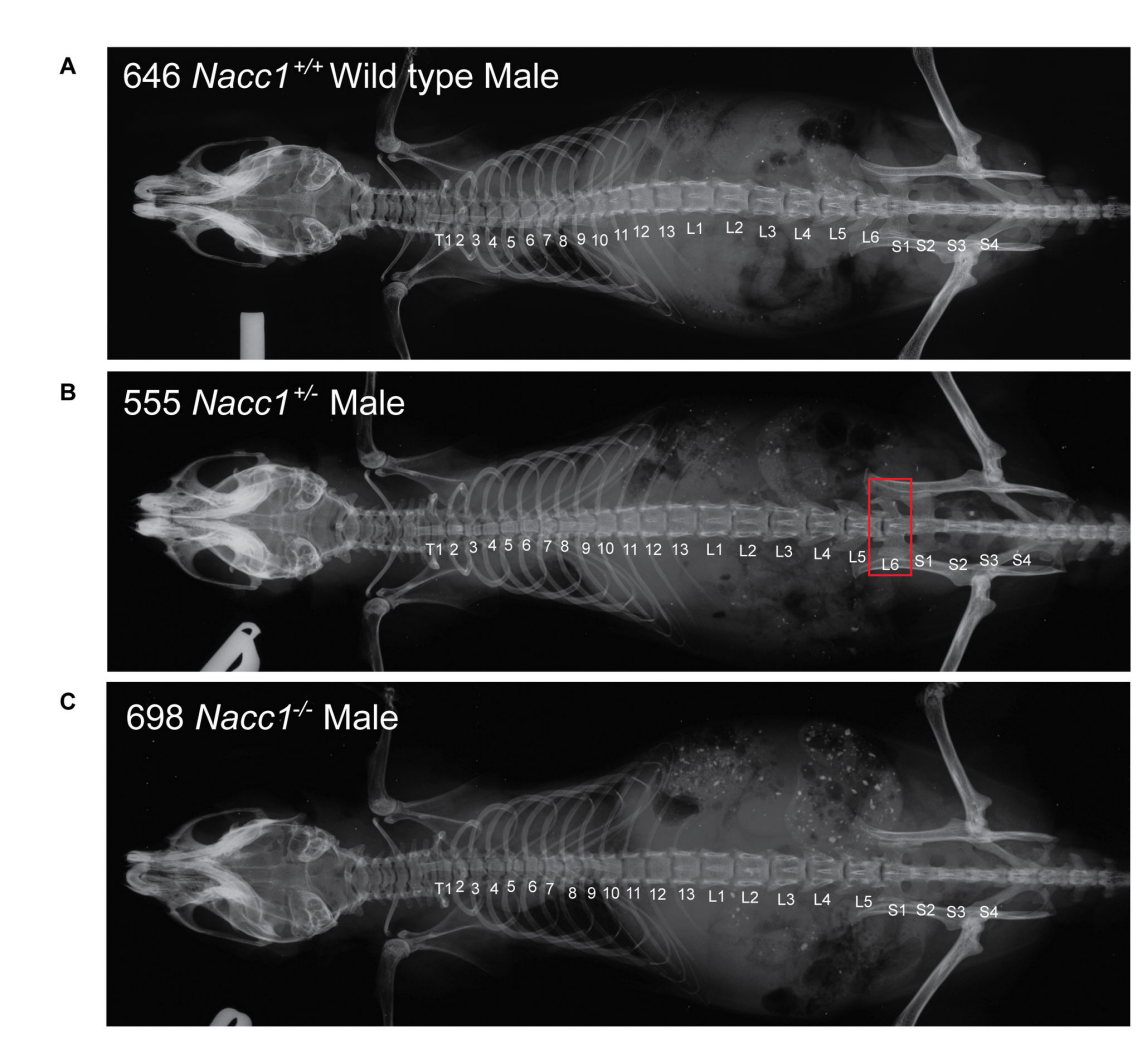
Reduced NAC1 expression alters axial skeletal patterning. The images show representative whole-body radiographs comparing the skeletal organization of adult Nacc1^+/+^ (wild type, **A**), Nacc1^+/-^ (heterozygous, **B**), and Nacc1^-/-^ (knockout, **C**) mice. The major defects are an absence of the sixth lumbar vertebra (L6) in the Nacc1^-/-^ mouse and an intermediate phenotype showing partial transformation of L6 into a sacral identity (**B**, red box). The numbers denote the number of vertebrae in the thoracic (T), lumbar (L), and sacral (S) regions of the vertebral column.

**Figure 3:**
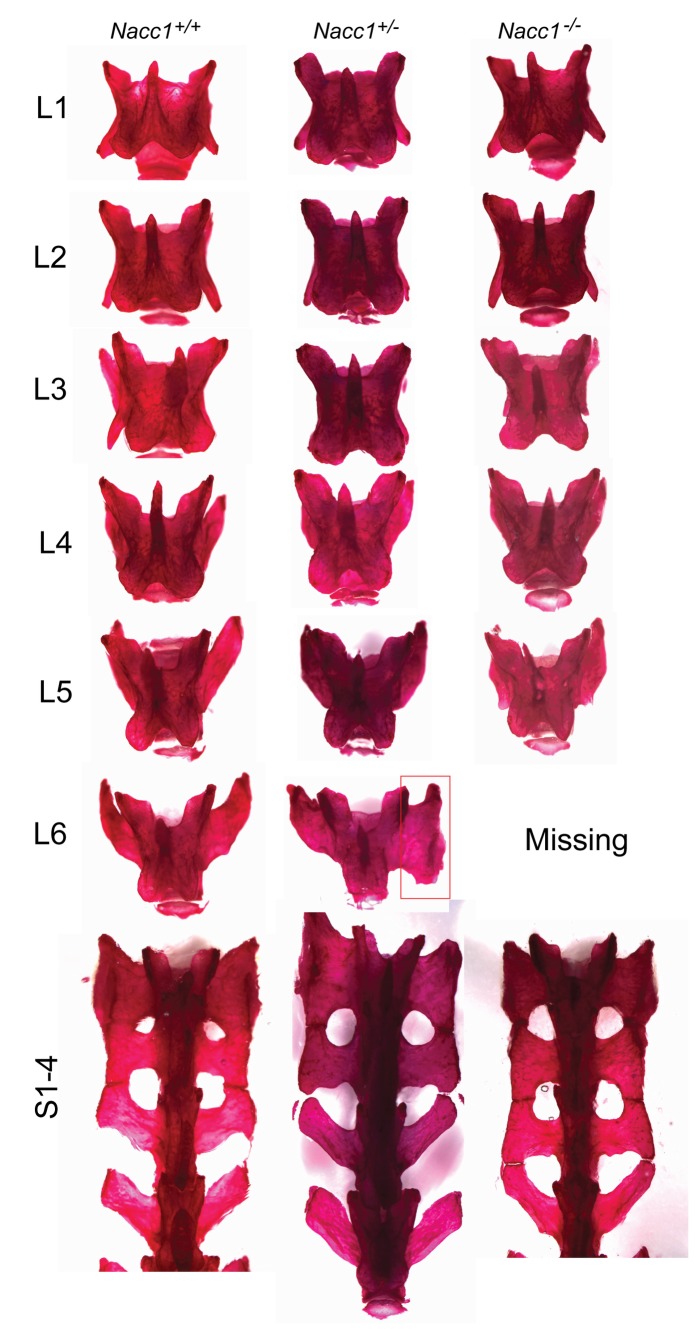
Reduced NAC1 expression ablates or changes the conformation of the sixth lumbar vertebra 6 (L6). Comparison of representative Alizarin red-stained lumbar (L) and sacral (S) vertebrae of adult Nacc1^+/+^ (wild type), Nacc1^+/-^ (heterozygous), and Nacc1^-/-^ (knockout) mice. In the Nacc1^-/-^ animal, L6 is missing, and the processes of all sacral vertebrae (S1-4) are fused; indeed, caudal extensions of the broad wings of S3 in this genotype resemble the profile of vertebra S2 rather than the usual contours of S3 in Nacc1^+/+^ mice. In the Nacc1^+/-^ counterpart, L6 has undergone partial (unilateral) transformation into a sacral identity (“sacralization,” red box). The lumbar vertebrae were disarticulated to better showcase their characteristic shapes.

The murine vertebral axis contains four regions, typically consisting of seven cervical (C), thirteen thoracic (T), six lumbar (L), and four sacral (S) vertebrae (formula: C7/T13/L6/S4). Radiographs of C57BL/6 mice showed that a deficiency of NAC1 expression correlated with a reduction in the number of pre-sacral vertebral elements (Figure 2). Careful examination demonstrated a patterning defect of the axial skeleton that occurred more frequently (Fisher’s exact test conducted by Graphpad software, ***p < 0.001) in Nacc1^-/-^ mice than their Nacc1^+/+^ littermates (Table 2). In particular, Nacc1^-/-^ mice presented without the sixth lumbar vertebra (L6) and with fusion of all sacral vertebrae (S1-4) rather than only the rostral two (S1-2) (Figure 3). This vertebral respecification displayed near complete penetrance (94.4%), resulting in nearly all knockout animals having either a T13/L5 (83.3% of affected individuals) or T12/L6 (11.1%) configuration (Table 2). In contrast, the normal arrangement in their Nacc1^+/+^ counterparts was T13/L6 (Figure 2); only 5.3% of wild type animals had the T13/L5 phenotype (which is within the normal variation for a wild type mouse population [28]). The L5 arrangement in animals of both knockout and wild type genotypes appeared to reflect a loss of L6 vertebra. Nonetheless, the extension of sacral identities one position forward along the vertebral axis resulted in a constant total number of pre-caudal vertebrae (Supporting Information, Figure S3). Thus, it seems likely that NAC1 may regulate vertebral identity at the lumbosacral transition. The vertebral morphology in Nacc1^-/-^ mice with the vertebral formula T12/L6 indicated that this finding resulted from conversion of the caudal thoracic vertebra (T13) into a new lumbar vertebra coupled with the expected loss of L6. This finding implicated a role for NAC1 in specifying skeletal identities at the thoracolumbar junction.

**Table 2:** Axial skeletal phenotypes of the respective *Nacc1* genotypes.

**Genotype**	***Nacc1*^*+/+*^**	***Nacc1*^*+/-*^**	***Nacc1*^*-/-*^**	
**Vertebral Pattern**				
**T14L6**	1 (5.3%)			
**T13L6**	16 (84.2%)	8 (38.1%)	1 (5.6%)	
**T13L6sacralized**	1 (5.3%)	7 (33.3%)		
**T12L6**			2 (11.1%)	
**T13L5**	1 (5.3%)	6 (28.6%)	15 (83.3%)	Total
**Total**	19	21	18	58

Using Fisher’s exact test, p 1.006991x10^-08^, indicating the above mentioned observed differences were statistically significant.

The Nacc1^+/-^ mice adopted an intermediate phenotype relative to their Nacc1^-/-^ and Nacc1^+/+^ littermates, a result suggesting that specification of the thoracolumbar and lumbosacral junctions depended on the expression levels of NAC1 (Figures 2 and 3). We found that 28.6% of Nacc1^+/-^ mice had an altered T13/L5 phenotype, 38.1% of them followed the usual Nacc1^+/+^ T13/L6 configuration, and 33.3% of them had an asymmetrical partial transformation of vertebra L6 into a sacral identity (T13/L6 sacralized) (Table 2). This finding presented as either the left or right process of the L6 vertebra adopting the butterfly-wing contours characteristic of the S1 vertebra (red boxes in Figure 2, middle panel, and Figure 3), thereby giving rise to an asymmetric vertebral axis.

In contrast to the altered thoracolumbar skeleton, mice with different *Nacc1* genotypes had regionally identical morphological features in the cervical (Supporting Information Figure S1), rostral thoracic (i.e., T1-9; Supporting Information Figure S2), and tail (Supporting Information Figure S3) vertebrae. This similarity implies that NAC1 expression has little if any responsibility for determining the fate of vertebral progenitors in the most rostral and caudal portions of the vertebral column, especially at the cervico-thoracic and sacro-caudal transitions.

**Figure 4:**
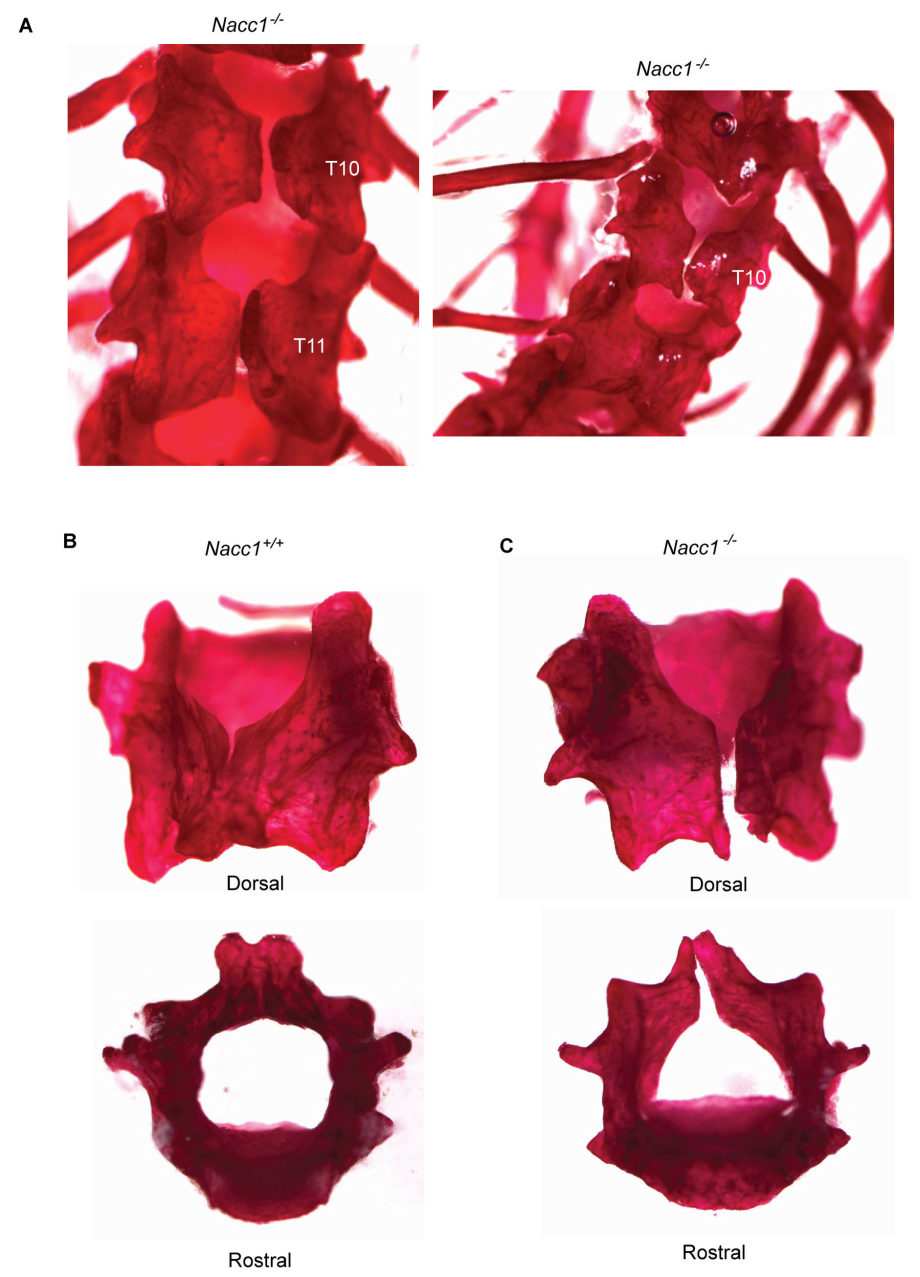
Loss of NAC1 expression is associated with dorsal non-closure of the vertebral arches in thoracic vertebrae 10 and 11. A. Vertebral fissures affecting adult, male (left) and female (right) Nacc1^-/-^ mice at T10 and T11. Disarticulated, Alizarin red-stained T10 vertebrae from a Nacc1^+/+^ (B) and Nacc1^-/-^ (C) demonstrating the presence of an observable dorsal fissure in the null mutant animals.

Examination of the thoracic vertebrae in Nacc1^-/-^ mice revealed a significantly higher incidence of fissures (66.7%) affecting the dorsal surface of vertebrae T10, T11, and T12 as compared to the incidence observed in wild type mice (6.7%) (Fisher’s exact test conducted by Graphpad software, **p <0.01) (Table 3 Figure 4, and Supporting Information Figure S2). The above findings suggest that NAC1 also regulated region-specific bone development of caudal thoracic vertebrae in addition to its function in specifying skeletal fate at the thoracolumbar junction.

**Table 3:** Frequency of vertebral non-closure in the respective *Nacc1* genotype.

**Genotype**	***Nacc1*^*+/+*^**	**Nacc1^+/-^**	**Nacc1^-/-^**	
**Presence of Fissure**	1 (6.7%)	6 (33.3%)	8 (66.7%)	
**Absence of Fissure**	14 (93.3%)	12 (66.7%)	4 (33.3%)	**Total**
**Total**	15	18	12	45

Using Fisher’s Exact test, p 0.004658915, indicating the observed differences were statistically significant.

**Figure 5:**
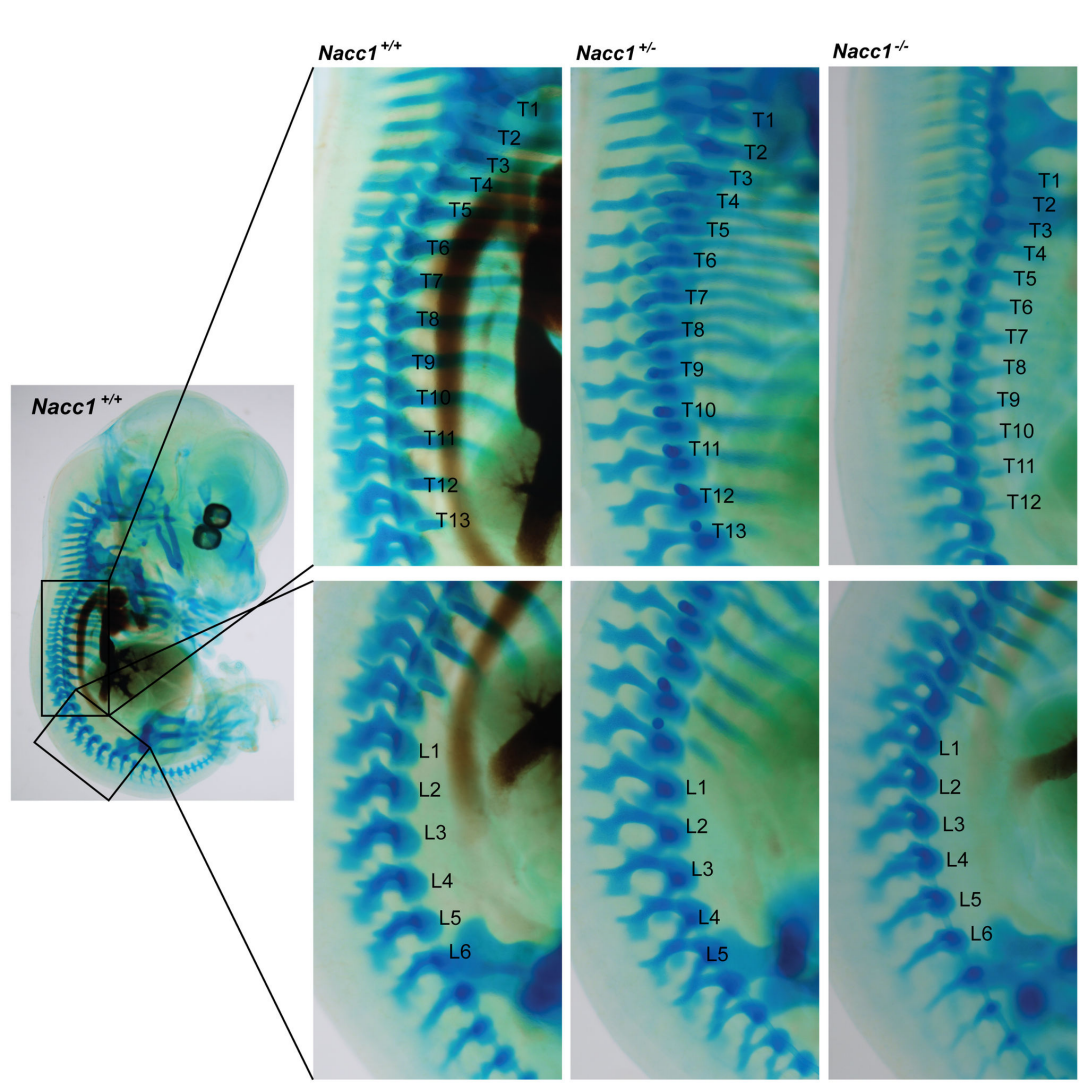
Reduced NAC1 expression is associated with altered specification of cartilage primordia for vertebral patterning. Comparison of representative Alcian blue-stained, whole mount mouse embryos at embryonic day 15 (E15) reveals the typical vertebral formulas related to the Nacc1^+/+^ (wild type, formula T13/L6), Nacc1^+/-^ (heterozygous, formula T13/L5), and Nacc1^-/-^ (knockout, formula T12/L6) genotypes. The numbers denote the number of vertebrae in the thoracic (T, top row) and lumbar (L, bottom row) regions of the vertebral column.

To explore when the vertebral patterning defect in adult Nacc1^-/-^ mice was initiated during development, we collected E15 mouse embryos (i.e., just after conclusion of organogenesis) that had undergone sufficient development of cartilaginous structures to permit visualization by Alcian blue staining (Figure 5 and Supporting Information Figure S4). While the Nacc1^+/+^ embryos had the expected number and patterning of the vertebral column (i.e., T13/L6), both Nacc1^+/-^ and Nacc1^-/-^ embryos demonstrated a missing pre-sacral vertebra (i.e., either T13/L5 or T12/L6) in many individuals (Table 4 and Figure 5). These data indicated that NAC1 acts sometime prior to the end of mesenchymal fate specification during organogenesis to control the fate of vertebral anlagen.

**Table 4:** Vertebral phenotypes of (E15) embryos in a single litter.

**Embryo**	**Genotype**	**Vertebral number**
A	Nacc1^-/-^	T12L6
B	*Nacc1* ^*+/+*^	T13L6
C	Nacc1^+/-^	T13L5
D	Nacc1^+/+^	T13L6
E	Nacc1^+/-^	T13L5
F	Nacc1^-/-^	T12L6
G	Nacc1^-/-^	T13L5
H	Nacc1^-/-^	T12L6

### Widespread NAC1 expression in embryonic mice suggests an important role in the genesis of many axial organs and tissues

**Figure 6:**
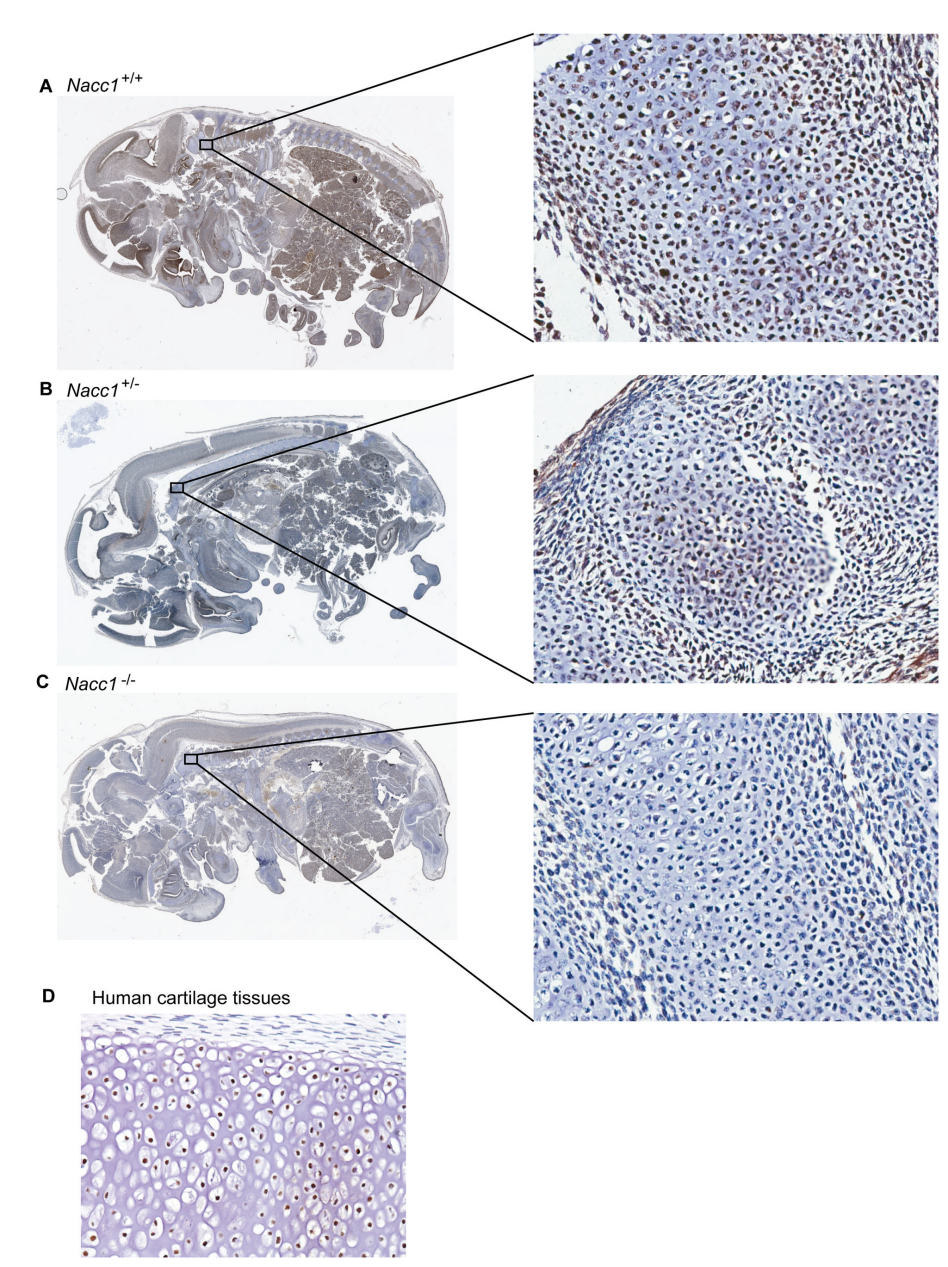
NAC1 expression in the chondrocytes of wild type mouse embryos. Comparison of representative para-sagittal sections of Nacc1^+/+^ (wild type, **A**), Nacc1^+/-^ (heterozygous, **B**), and Nacc1^-/-^ (knockout, **C**) mouse embryos at embryonic day 16 (E16) shows the widespread distribution of NAC1 in various tissues, including chondrocytes (boxed regions). The wild type expression pattern exhibits widespread immunoreactivity including such axial tissues as vertebral primordia and the peri-ventricular germinal zones in the brain. **D**. Chondrocytes in cartilaginous tissues of a human embryo (estimated gestational age, 7 weeks) also exhibit extensive NAC1 immunoreactivity. Anti-NAC1 immunohistochemistry (using species-specific primary antibodies) with hematoxylin as the counterstain.

To assess the possible roles of *Nacc1* in mouse embryonic development, we examined the expression of NAC1 in the tissues of mouse E16 embryos. NAC1 immunoreactivity in Nacc1^+/+^ embryos was observed in the nuclei of many cells within the CNS (especially the periventricular germinal layers) and peripheral nervous system (PNS), the epithelium lining the gastrointestinal and respiratory tracts, and in chondrocytes within bony anlagen (Figure 6A). In contrast, NAC1 immunoreactivity was less widespread and intense in age-matched littermate Nacc1^+/-^ embryos (Figure 6B), and was undetectable in *Nacc1* ^*-/-*^ embryos (Figure 6C). Similar to mouse embryonic chondrocytes, NAC1 also was strongly expressed in human embryonic chondrocytes (Figure 6D). These findings indicated that NAC1 expression during the period of axial skeletal specification and differentiation was essential for both murine and human chondrocytes. Furthermore, the pervasive presence of NAC1 in the stem cell zones within the CNS suggests the potential for this protein to regulate the fate of cells within other axial systems as well, including the CNS and gastrointestinal tract.

### Diminished NAC1 expression reduced chondrocyte motility and proliferation

We established primary costal chondrocyte cultures from P1 neonates (Supporting Information Fig. S5A) to compare ability of cell migration and proliferation among cells obtained from different *Nacc1* genotypes. As expected, the isolated Nacc1^+/+^ cells expressed the highest amount of NAC1 protein followed by Nacc1^+/-^ cells while Nacc1^-/-^ cells did not express any detectable level of NAC1 (Supporting Information Fig. S5B). The identity of chondrocytes was confirmed by their characteristic cobblestone-like appearance (Supporting Information Fig. S5C), positive Alcian blue staining (Supporting Information Fig. S5D), and the expression of a panel of chondrocyte markers including high levels of aggrecan and collagen type 2 together with low levels of collagen type I (Supporting Information Fig. S5E) [25]. Using chondrocytes derived from the coastal cartilage is relevant, as we found that some of the Nacc1^-/-^ mice with the T12 phenotype had a missing pair of ribs.

**Figure 7:**
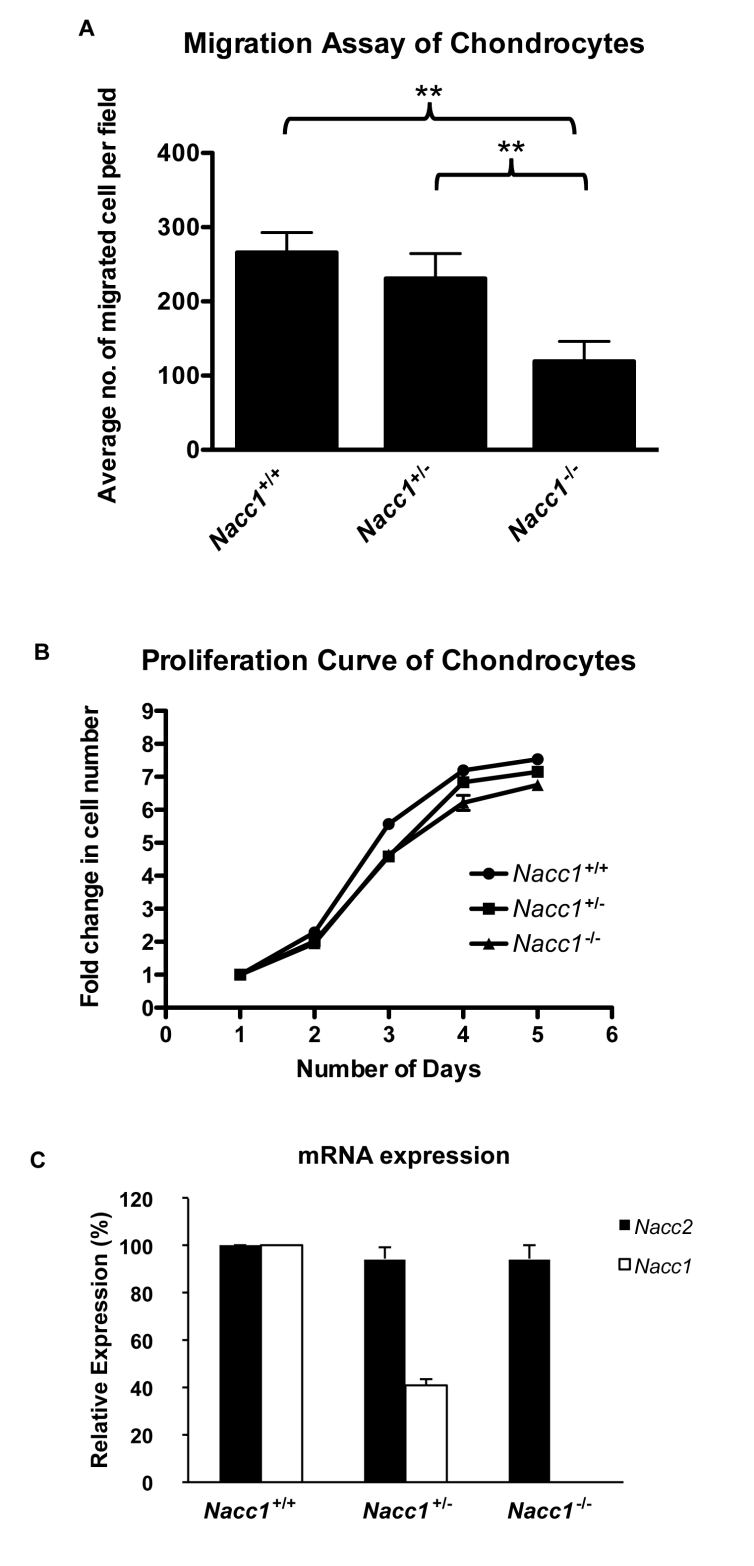
Reduced NAC1 expression decreases chondrocyte migration but does not affect chondrocyte proliferation or the balance of NAC2 expression. Comparison of cultured costal chondrocytes derived from Nacc1^+/+^ (wild type), Nacc1^+/-^ (heterozygous), and Nacc1^-/-^ (knockout) mouse pups at postnatal day 1 (P1) with respect to their migratory (**A**) and proliferative (**B**) properties and their complements of NAC1 and NAC2 (a protein closely related to NAC1, **C**). **A**: ** denotes a significant difference in cell migration, p < 0.01). **B**: *Nacc1* depletion did not affect chondrocyte division. **C**: Real-time quantitative PCR analysis showed that levels of *Nacc2* in chondrocytes of Nacc1^-/-^ mice were not altered to compensate for the reduction in *Nacc1*.

As NAC1 has been found to be an actin-binding protein and has been reported to affect cellular motility [18,19], we compared the migration of Nacc1^-/-^ chondrocytes to those of Nacc1^+/-^ and Nacc1^+/+^ chondrocytes. The number of chondrocytes crossing a porous membrane was significantly decreased in *Nacc1* ^*-/-*^ cells as compared to the *Nacc1* ^*+/-*^ and *Nacc1* ^*+/+*^ cells (ANOVA test, *p < 0.05) (Figure 7A). Post-hoc statistical analysis further indicated that the significant difference in chondrocyte migration occurred not only between *Nacc1* ^*+/+*^ and Nacc1^-/-^ cells but also between Nacc1^+/-^ and Nacc1^-/-^ cells (Tukey test, **p < 0.01). In contrast, the number of migrated cells of *Nacc1* ^*+/+*^ and *Nacc1* ^*+/-*^cells were comparable (Tukey test). Taken together, these results indicate that the migratory capacity of chondrocytes declines if their NAC1 expression is reduced.

To exclude the possibility that higher proliferation rates of *Nacc1* ^*+/+*^ chondrocytes contributed to the increased number of migrating cells, we conducted a proliferation assay to determine the growth rates of chondrocytes isolated from the different *Nacc1* genotypes. We found that the proliferative activities of chondrocytes were independent of their *Nacc1* genotypes, particularly during the time frame (15 hrs. overnight between days 0 and 1) used in the migration assay (Figure 7B).

Given the homology in protein sequence between NAC1 and NAC2 (NCBI Genbank), it is possible that NAC2 may complement NAC1 deletion functions in mouse chondrocytes. Thus, we also determined the mRNA expression levels of *Nacc2*. We demonstrated that *Nacc2* expression levels in Nacc1^-/-^ cells remained the same as Nacc1^+/+^ cells (Figure 7C). This result implies that a compensatory increase in NAC2 expression does not take place in NAC1 depleted cells.

### Reduced NAC1 production altered expression of matrilin-3 and matrilin-4 in *Nacc1* ^*+/+*^ versus Nacc1^-/-^ chondrocytes

**Figure 8:**
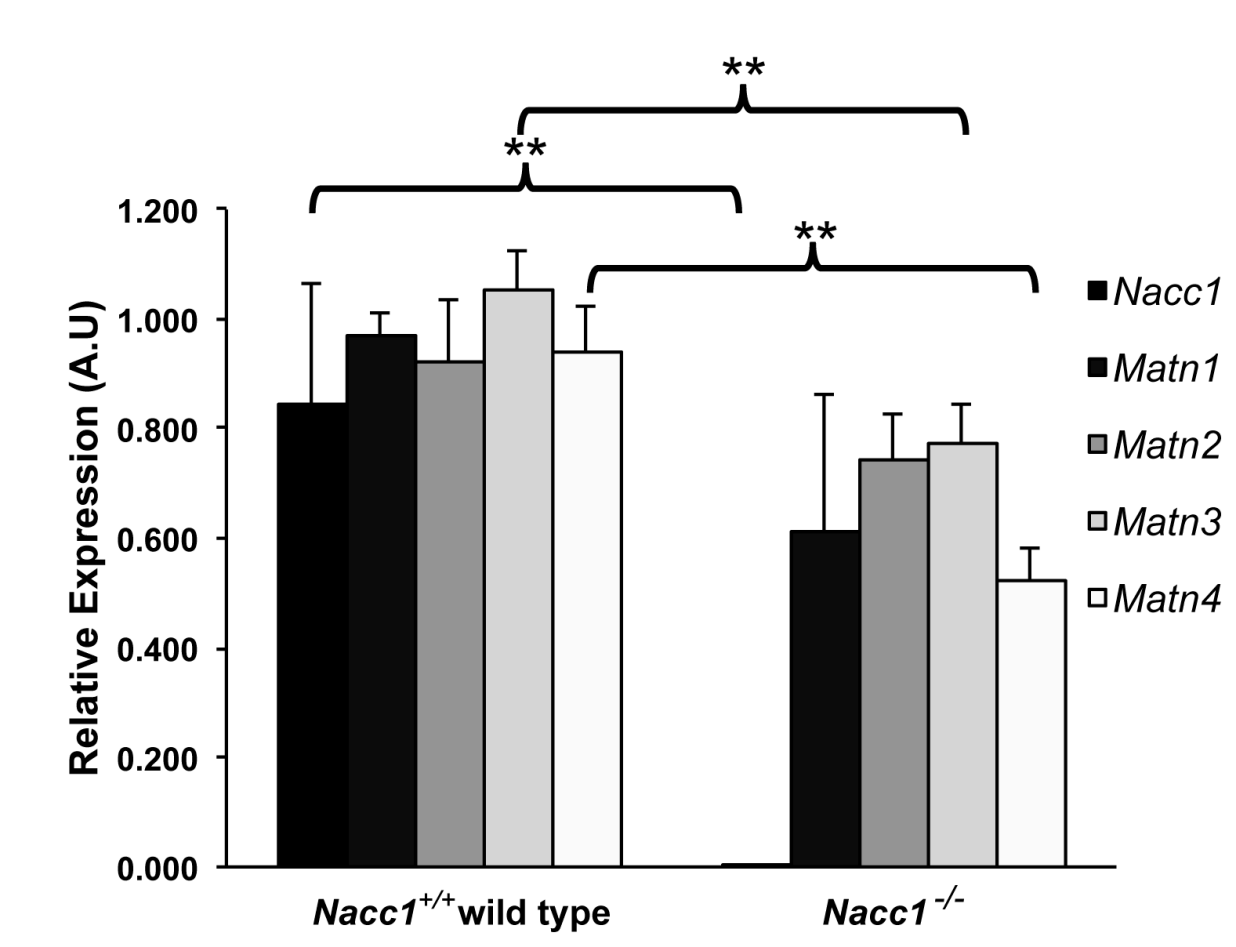
Reduced NAC1 expression is associated with alterations in chondrocytic expression of *matrilin-3* and *matrilin-4*. Quantitative real-time PCR analysis performed on cultured costal chondrocytes derived from Nacc1^+/+^ (wild type) and Nacc1^-/-^ (knockout) mouse pups at postnatal day 1 (P1) to measure the relative expression levels of the different matrilin (MATN) proteins. Only MATN3 and MATN4 are expressed at a significantly different level (** denotes p < 0.01) between the two genotypes.

To identify genes and pathways that may be associated with NAC1 expression in mice, we conducted a preliminary study in which we interrogated the NAC1-regulated transcriptome in different types of normal mouse tissues including cartilage using a microarray. We accomplished that by comparing the gene expression of chondrocytes cultured from the coastal cartilage of Nacc1^-/-^ and Nacc1^+/+^ mice. Again, we used this source of chondrocytes because it enabled us to collect a sufficient number of chondrocytes for the study. The chondrocytes derived from the two wild types and three homozygous knockout animals showed very consistent expression levels for the same genes. Based on our microarray studies, we found that matrilin4 was the only cartilage gene that was significantly regulated by NAC1. As the matrilin family consists of 4 distinct family members, we analyzed their expression patterns using quantitative real-time PCR. As a result, we found that in addition to matrilin4, matrilin3 was also upregulated by NAC1 (Student t-test, *p<0.05 and **p<0.01 for MATN3 and MATN4, respectively) (Figure 8). Though the changes in matrilin3 and 4 expression may be modest, they are consistent in independent biological replicates. In contrast, the other two related matrilin genes, *Matn1* and *Matn2*, did not show significant difference in their expression between Nacc1^-/-^ and Nacc1^+/+^ chondrocytes (p>0.05). The results of the microarray analysis are shown in Supporting Information file 6.

In this study, we did not attempt to include NAC1^+/-^ heterozygous mice in gene expression study because defining the phenotype of the heterozygous animals appeared highly challenging. The intermediate phenotype of the heterozygous mice did not occur in all animals. According to table 2, one third of heterozygous mice exhibited the intermediate phenotype of a partially sacralized L6, while 28.6% of heterozygous animals show complete sacralization of the L6, leading to a T13L5 phenotype. On the other hand, 38.1% of heterozygous animals have the distinctive wild type configuration of T13L6. The incomplete penetrance of the phenotype in the heterozygous animals prompted us to exclude heterozygous animals from microarray studies.

## Discussion

NAC1 has emerged as a critical molecule that participates in several biological and pathological processes, including addiction to psychostimulants [2,3] and cancer aggressiveness [8–16]. By comparing phenotypes between Nacc1^-/-^ (knockout) and Nacc1^+/+^ (wild type) mice, we sought to elucidate the role(s) of *Nacc1* in regulating embryonic development and tissue homeostasis. In this report, we demonstrated that the loss of NAC1 expression was compatible with survival and normal development in most mice, although fewer Nacc1^-/-^ newborns survived until weaning. The most remarkable phenotypes associated with the mice with the Nacc1^-/-^ genotype involved the axial skeleton, and included a defect affecting vertebral specification of the thoracolumbar and lumbosacral boundaries as well as frequent non-closure of the dorsal arches of the T10/T11 vertebrae. Chondrocytes express abundant NAC1 in Nacc1^+/+^ but not in Nacc1^-/-^ mouse embryos. The reduced *Nacc1* expression in chondrocytes decreased cell migratory potential during development probably through altered extracellular matrix production. The above phenotypes were identifiable as early as E16 in Nacc1^-/-^ embryos and persisted until at least P1. Sacralization of L6 was also observed in Nacc1^+/-^ littermates, although to a lesser frequency than the null mutant animals. These findings have several implications regarding the biological roles of NAC1 in tissue homeostasis.

The results from this study suggest several potential mechanisms contributing to the abnormal phenotypes. One possibility is the reduced migratory capability of Nacc1^-/-^ chondrocytes. It has been established that chondromesenchymal cells condense and differentiate into chondrocytes, forming a soft cartilaginous model of the skeleton that is subsequently transformed into a calcified bony version. Morphogenesis of the vertebral column largely depends on the initial formation of cartilage, and the subsequent remodeling of cartilage primordia into bones and joints. Thus, the decreased migration associated with Nacc1^-/-^ chondrocytes may be responsible for the insufficient migration of precursor cells (i.e., chondromesenchymal cells) into somites, where mesenchymal (including chondromesenchymal) cells condense and divide the axis of vertebrate embryos into segments. Somitogenesis, or the process of somite formation, depends on segment-specific cell differentiation and organ patterning; somite remodeling during organogenesis leads to development and differentiation of mature bony vertebrae and their associated musculature [29]. As migration of cells has been demonstrated before somite segmentation [30], the defective migration of Nacc1^-/-^ chondrocytes is a reasonable explanation for the respecification of vertebral structures at the thoracolumbar and lumbosacral junctions as well as the dorsal midline fissures in the caudal few thoracic vertebrae. The non-closure of the vertebral elements in Nacc1^-/-^ mice is reminiscent of the spina bifida occulta, a birth defect in multiple species (including humans) that is characterized by a similar failure of the vertebrae to close at the midline. It has been reported that spina bifida-like disorders generally are associated with reduced migration and proliferation of mesenchymal chondrocyte precursor cells [31,32].

The molecular mechanisms underlying the reduced migration capacity of Nacc1^-/-^ chondrocytes might be multifaceted. For example, we have recently demonstrated that NAC1 is an actin-binding protein [18]. Actin is a critical element of the intracellular cytoskeleton that is essential for cell migration and proliferation [33]. Modulation of actin interactions that are essential for these processes may represent an important mechanism by which NAC1 controls patterning of the axial skeleton. In addition, NAC1 also appears to be a transcriptional co-regulator of other genes whose products are involved in cell movement. Transcriptome analysis between NAC1-expressing SKOV3 human ovarian cancer cells and NAC1-siRNA silenced SKOV3 cells demonstrated that several genes differentially expressed by NAC1 were involved in cell migration [34]. These include forkhead box Q1 (*FOXQ1*) [34], insulin-like growth factor-binding protein-6 (*IGFBP6*) [35], chemokine (C-X-C motif) ligand 1 (CXCL1, also known as GROα) [36], drebrin-like protein (*DBNL*, also known as mammalian actin-binding protein-1) [37], and Rho-associated, coiled-coil-containing protein kinase 2 (ROCK2) [38]. Further work will be required to define the precise nature of the signaling cascades linking NAC1 to these genes.

Besides the reduced ability in cellular migration, defect in generation of cartilage matrix of Nacc1^-/-^ chondrocytes may serve as another mechanism attributing to axial skeletal defects in *Nacc1* null mice. In this study, we found that expression of matrilin-3 and matrilin-4 were significantly reduced in Nacc1^-/-^ chondrocytes as compared to their Nacc1^+/+^ counterparts. Matrilins are a group of non-collagenous extracellular matrix proteins that mediate interactions between collagen fibrils and other matrix proteins [39], and participate in the formation of fibrillar or filamentous extracellular matrix. These molecules are known to be important mediators of cell-matrix and matrix-matrix interactions, thereby providing a matrix permissible for chondrocyte differentiation and ossification during embryogenesis [40]. Interestingly, matrilin-3 is known for being expressed specifically in developing cartilage primordia [41]. Although the function of matrilin-4 is less well defined [42], it has also recently been demonstrated to be involved in cartilage development [43]. The association of NAC1 status with the extent of matrilin expression further supports a critical role for NAC1 in regulating chondrocyte development.

It has been established that expression of the Hox gene families plays an important role in vertebral patterning. For example, the vertebral patterning in the lumbar and sacral region is attributed to the Hox10 paralogous family of genes [44,45]. However, in our study on *Nacc1* mice, we found no evidence of involvement of Hoxc10 [44]. We measured the expression of Hoxc10 by quantitative real-time PCR in the chondrocytes derived from P1 pups that were Nacc1^-/-^, Nacc1^+/-^ and *Nacc1* WT, and found that Hoxc10 expression was not significantly associated with NAC1 genotype. Future studies are required to further investigate whether there is a shift in the Hox genes boundaries due to Nac1 status. Interestingly, it has been demonstrated that NAC1 interacts with nuclear transcription factors like Nanog [7], a homeobox (Hox) gene that helps maintain the pluripotency of mouse stem cells [6]. It is noteworthy that NAC1 expression in mouse embryos was particularly high in the peri-ventricular germinal cell zones of the brain where neural stem cells reside. Axial patterning and somite specification in the brain and vertebral column are known to be controlled by differential expression of multiple Hox genes [46]. Additional study will be necessary to understand why reduced NAC1 leads to subtle vertebral defects limited to only discrete vertebrae and in the apparent absence of abnormalities in the central nervous system.

In conclusion, Nacc1^-/-^ mice are characterized by a lower survival rate for embryos or newborns, and those survived often develop subtle but reproducible gross abnormalities in particular thoracic and lumbar vertebrae. NAC1 is expressed at high levels in developing wild type cartilage, and cultured chondrocytes obtained from *Nacc1* ^*-/-*^ mice exhibit significant reductions in cellular migration and the expression of matrilin-3 and 4, which together may account for the defective vertebral patterning. Our data indicate biological functions of *Nacc1* in murine tissue development, and the findings are fundamental for future studies focusing on pathobiological processes involving NAC1. 

## Supporting Information

File S1 Microarray expression data comparing Nacc1^+/+^ *vs*. Nacc1^-/-^ chondrocytes. (XLSX)

Figure S1 Cervical vertebrae of Nacc1^+/+^ wild type *vs*. Nacc1^-/-^ mice. (TIF)

Figure S2 Dorsal aspect of thoracic vertebrae of Nacc1^+/+^ wild type *vs*. Nacc1^-/-^ mice. (TIF)

Figure S3 Post-sacral vertebral regions of the respective *Nacc1* genotypes. (TIF)

Figure S4 Comparison of representative Alcian blue-stained, whole mount mouse embryos at embryonic day 15 (E15). (TIF)

Figure S5 Characterization of cultured costal chondrocytes derived from neonatal mice. **A**. Genotyping for a representative litter of postnatal day 1 (P1) mice born to a Nacc1+/- (heterozygous) breeding pair, showing the Nacc1 genotypes (+/+ = wild type, +/- = heterozygous knockout, -/- = homozygous knockout) of seven pups (individually identified using letters) by gel electrophoresis of the PCR products. **B**. Western blot analysis of NAC1 expression in cultured chondrocytes from representative individuals (identified by the letter in parentheses) exhibiting the dependence of protein expression on the number of wild-type Nacc1 alleles. Gapdh = glyceraldehyde 3-phosphate dehydrogenase, a constitutively expressed housekeeping gene used to normalize the quantitative-PCR results. **C**. Morphology of cultured chondrocytes from representative individuals (identified by the letter in parentheses) demonstrating that cell morphology in vitro is comparable across all genotypes. Scale bar is 100 µM. **D**. Alcian blue staining of representative cultured chondrocytes from a Nacc1-/- animal showing that chondrocytes but not fibroblasts will be labeled using this cartilage marker. **E**. Real-time quantitative PCR analysis of mRNA expression for the chondrocyte markers aggrecan and collagen types I and II, showing the extent of marker expression relative to the housekeeping gene.
(TIF)
