# Supplementary figures and images for: Loss of NAC1 Expression Is Associated with Defective Bony Patterning in the Murine Vertebral Axis
Source: PLoS One. 2013 Jul 26;8(7):e69099. doi: 10.1371/journal.pone.0069099 (PMC3724875; doi:10.1371/journal.pone.0069099)

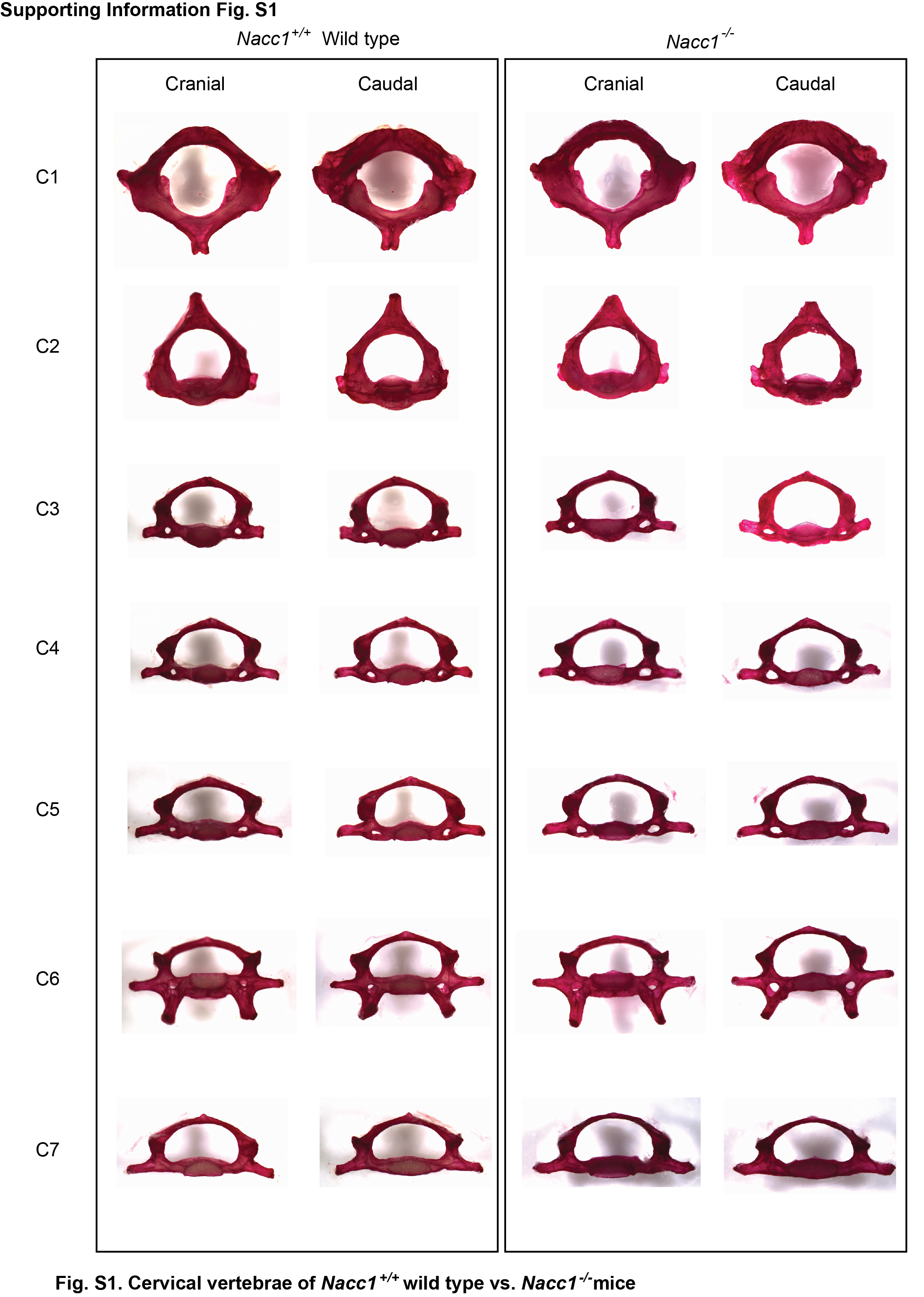

Supplement: Figure S1 — (TIF) [file pone.0069099.s002.tif]

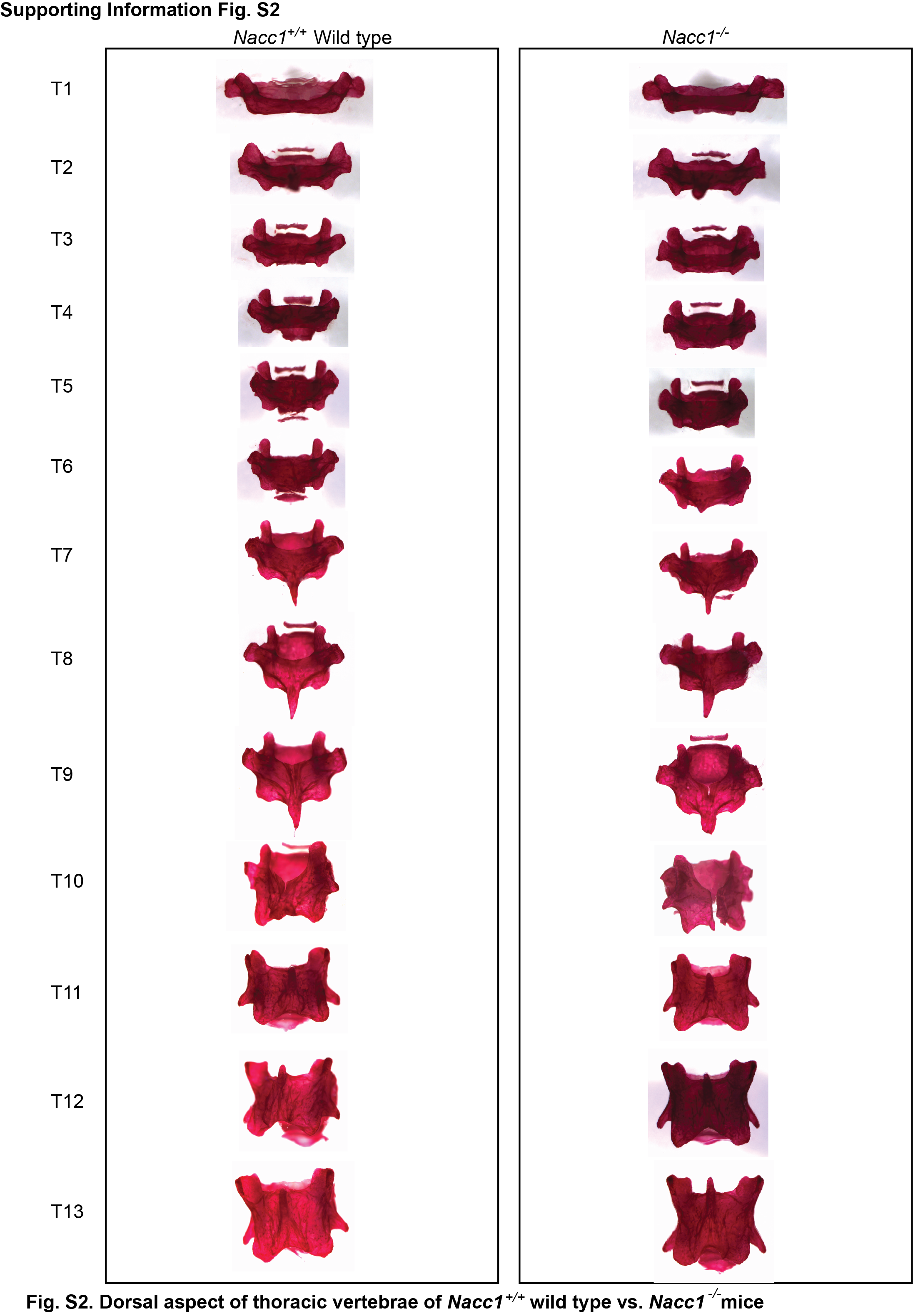

Supplement: Figure S2 — (TIF) [file pone.0069099.s003.tif]

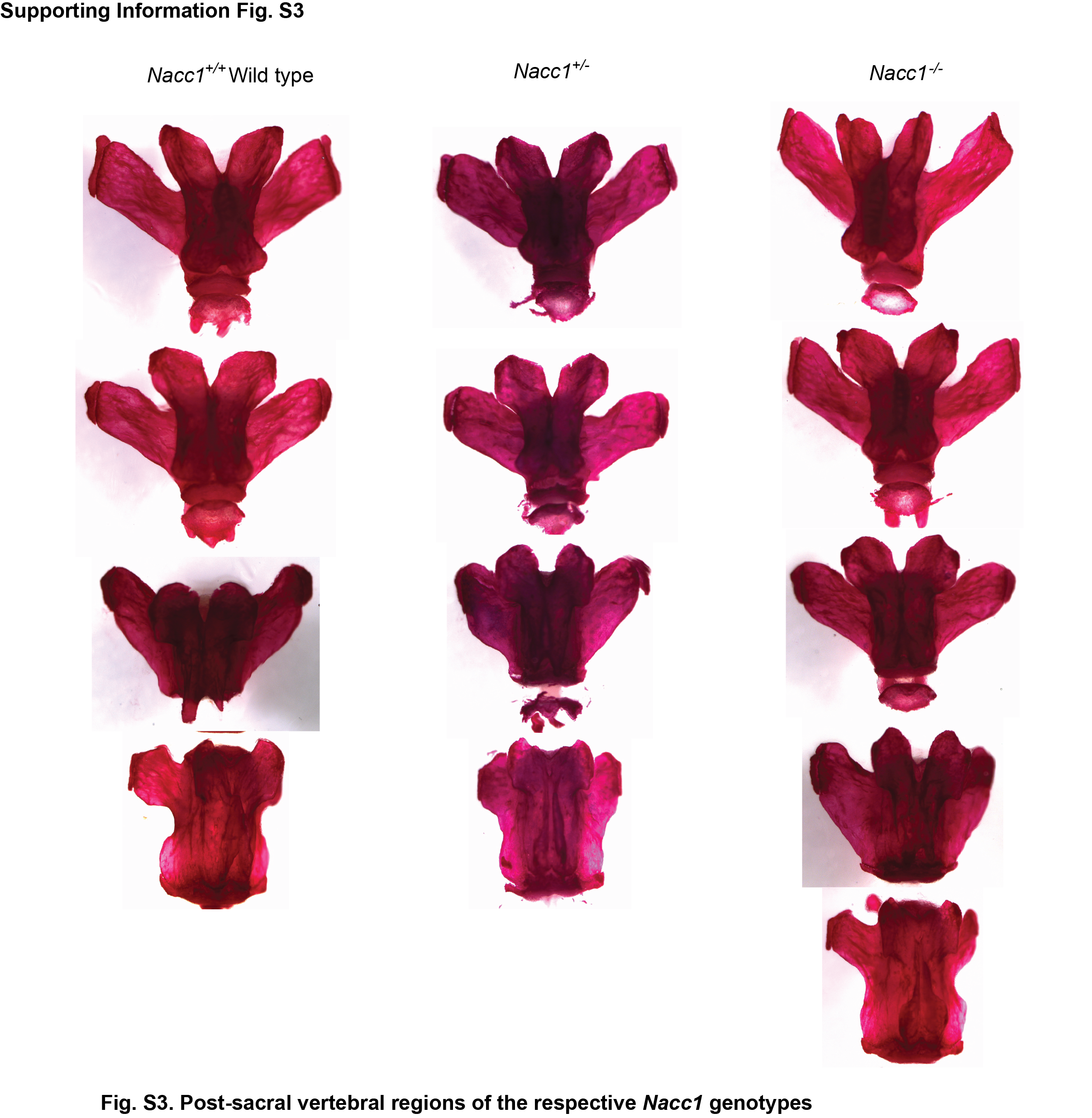

Supplement: Figure S3 — (TIF) [file pone.0069099.s004.tif]

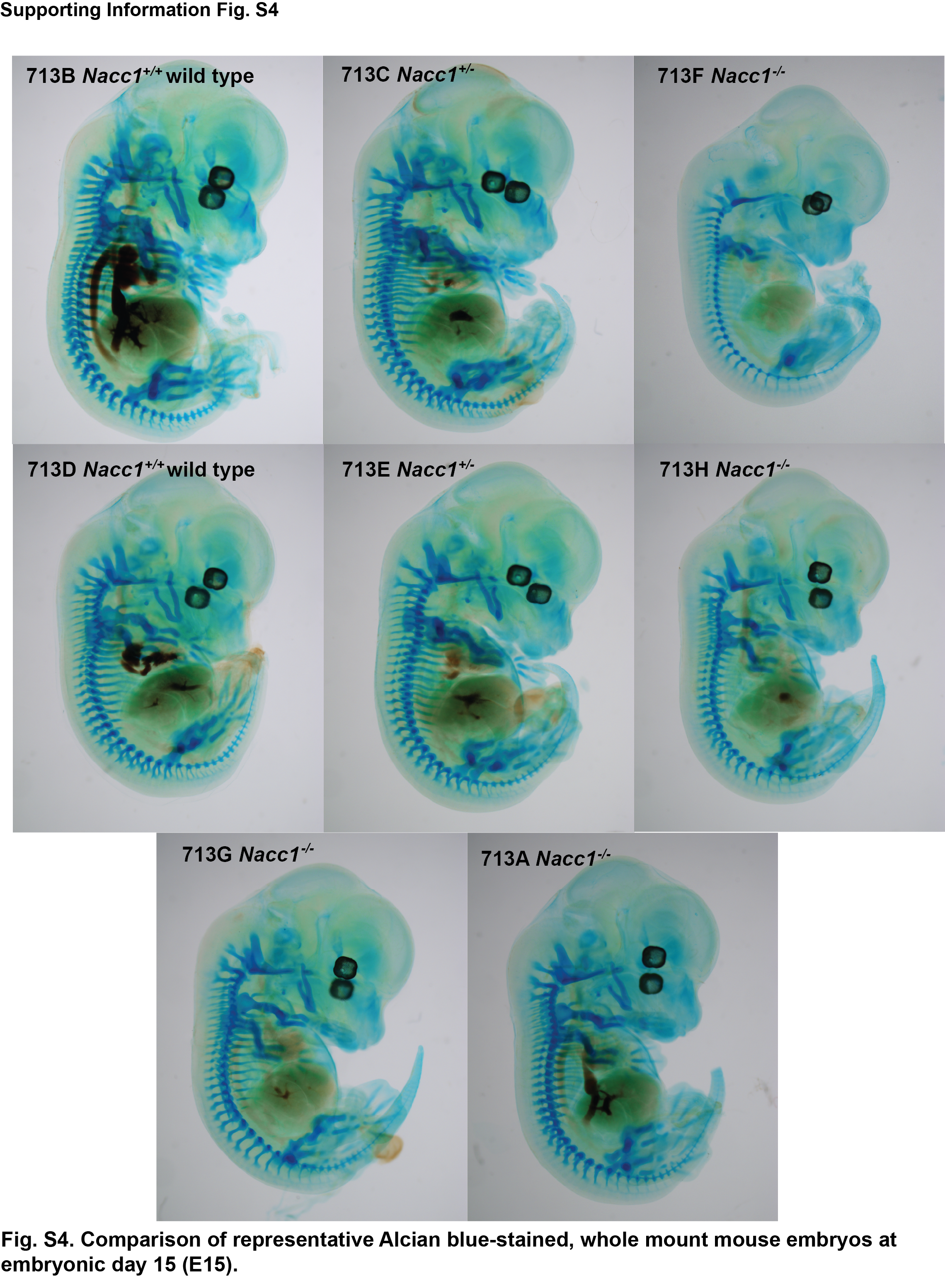

Supplement: Figure S4 — (TIF) [file pone.0069099.s005.tif]

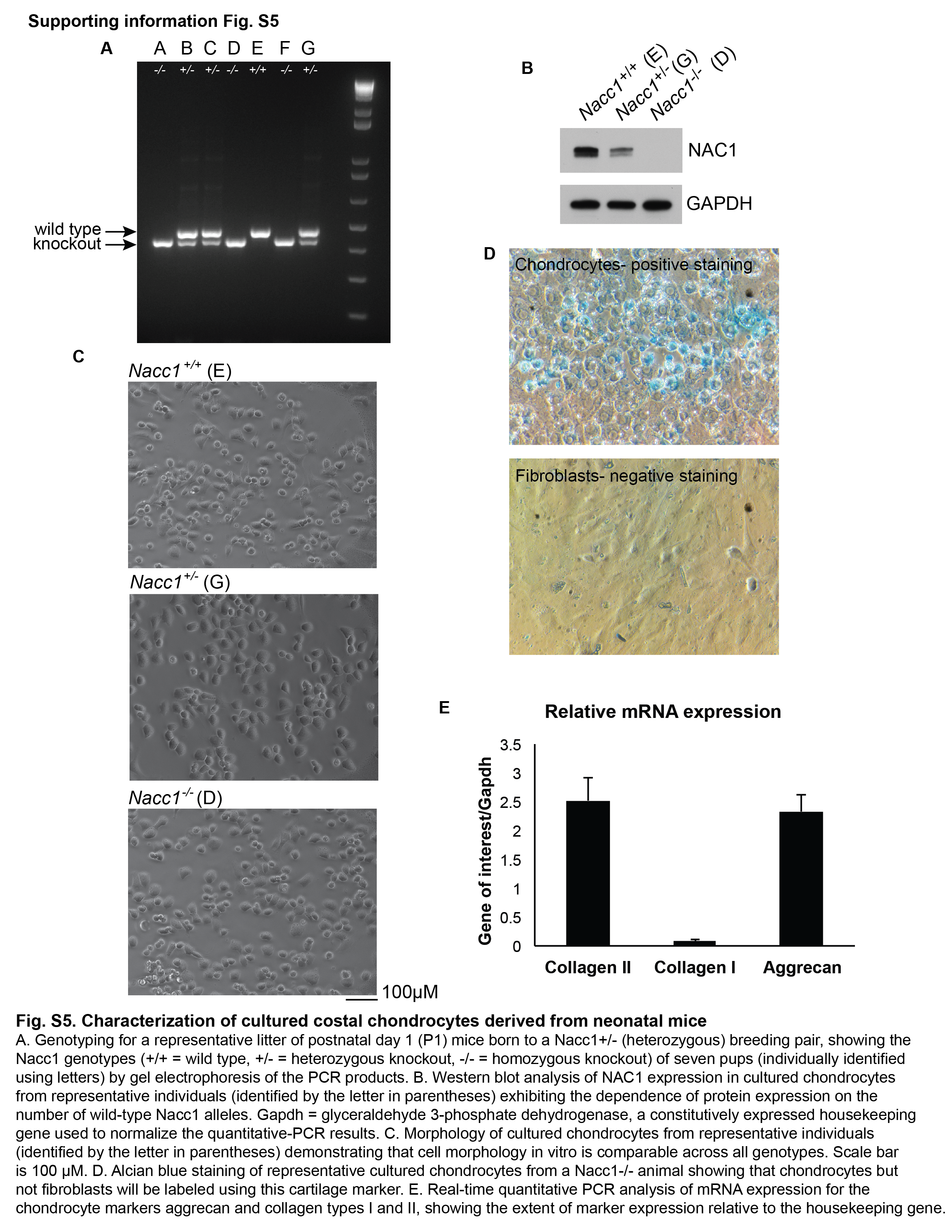

Supplement: Figure S5 — A. Genotyping for a representative litter of postnatal day 1 (P1) mice born to a Nacc1+/- (heterozygous) breeding pair, showing the Nacc1 genotypes (+/+ = wild type, +/- = heterozygous knockout, -/- = homozygous knockout) of seven pups (individually identified using letters) by gel electrophoresis of the PCR products. B. Western blot analysis of NAC1 expression in cultured chondrocytes from representative individuals (identified by the letter in parentheses) exhibiting the dependence of protein expression on the number of wild-type Nacc1 alleles. Gapdh = glyceraldehyde 3-phosphate dehydrogenase, a constitutively expressed housekeeping gene used to normalize the quantitative-PCR results. C. Morphology of cultured chondrocytes from representative individuals (identified by the letter in parentheses) demonstrating that cell morphology in vitro is comparable across all genotypes. Scale bar is 100 µM. D. Alcian blue staining of representative cultured chondrocytes from a Nacc1-/- animal showing that chondrocytes but not fibroblasts will be labeled using this cartilage marker. E. Real-time quantitative PCR analysis of mRNA expression for the chondrocyte markers aggrecan and collagen types I and II, showing the extent of marker expression relative to the housekeeping gene. (TIF) [file pone.0069099.s006.tif]
